# Human Endogenous Retrovirus-H Long Terminal Repeat-Associating Protein 2 (HHLA2) is a Novel Immune Checkpoint Protein in Lung Cancer which Predicts Survival

**DOI:** 10.31557/APJCP.2021.22.6.1883

**Published:** 2021-06

**Authors:** Mayada Saad Farrag, Eman Mohamad Ibrahim, Tamer A El-Hadidy, Mohamed Farouk Akl, Alyaa R Elsergany, Heba Wagih Abdelwahab

**Affiliations:** 1 *Department of Pathology, Faculty of Medicine, Port Said University, Port Said, Egypt. *; 2 *Department of Pathology, Faculty of Medicine, Mansoura University, Mansoura, Egypt. *; 3 *Department Chest Medicine, Faculty of Medicine, Mansoura University, Mansoura, Egypt. *; 4 *Department of Clinical Oncology & Nuclear Medicine, Faculty of Medicine, Mansoura University, Mansoura, Egypt. *; 5 *Department Internal Medicine, Oncology Center, Faculty of Medicine, Mansoura University, Mansoura, Egypt. *

**Keywords:** HHLA2, Immunotherapy, Lung cancer, Metastasis, PFS

## Abstract

**Background:**

Lung cancer is one of the most frequently diagnosed malignancies. Human endogenous retrovirus-H long terminal repeat-associating protein 2 (HHLA2) is a recently discovered ligand of the B7 family. Blocking this immune checkpoint has become an important treatment option for lung cancer.

**Methods:**

The study includes 62 biopsy specimens either bronchoscopic or CT-guided biopsies diagnosed as lung cancer in Hospitals of Faculty of Medicine, Mansoura University, Egypt during the period from 2016 to 2020. Immunohistochemical Staining for *HHLA2* and *EGFR* was performed. *HHLA2* expression was assessed in different pathological types of lung Cancer, and it was correlated with other clinicopathologic parameters and patient prognosis.

**Results:**

We found a significant association between *HHLA2* expression and metastasis. About 83% of patients presented with metastasis showed positive expression of *HHLA2* compared to 44.4% in patients with no metastasis (p=0.02). Also, results show significant mild positive correlation between expression of *HHLA2* and *EGFR* markers (p=0.045). The mean OS time in cases with positive *HHLA2* expression was nearly half that of patients with negative expression of the markers. However, this difference was not statistically significant. But, PFS of patients was significantly lower among the group with positive expression of *HHLA2* compared to the group with negative expression of *HHLA2* (p= 0.01).

**Conclusions:**

This study reports that recently discovered, *HHLA2* is over expressed in lung cancer associating with higher stage. It is also correlated with EGFR overexpression. *HHLA2* could serve as a predictor of progression and distant metastasis. Also, it has potential to be effective immune target in lung cancer immunotherapy such as checkpoint blockade and antibody-drug conjugate treatment.

## Introduction

Lung cancer is one of the most frequently diagnosed malignancies. It is the major cause of cancer related mortality in males and the second major cause of cancer related mortality in females all over the world (Torre et al., 2016). In Egypt, National Population-Based Cancer Registry Program 2008 - 2011 ranked lung cancer as the sixth most common cancer in both genders (Ibrahim et al., 2014). Pathological review of lung cancer tissues is fundamental in diagnosis, prognosis and treatment decision of these cancers. Recent advances in genomic profiling identify new targetable oncogenes in lung cancer. Understanding the heterogeneous tumor microenvironment can open new avenues for better management (Chen et al., 2014).

The value of immunotherapy is recently emphasized in different tumors (Chang et al., 2019). Blocking the immune checkpoint (ICP) has become an important treatment option in the management of lung cancer. Programmed death ligand-1 (PDL-1) inhibitors are the first used ICP inhibitor. They have been approved in the United States and Europe as second-line therapy for metastatic NSCLCs (Zago et al., 2016). However, there are challenges to immunotherapy including immune-related toxicity and resistance to immunotherapy. This would cause more difficulties on tailoring treatment protocols (Zhang et al., 2019).

Human endogenous retrovirus-H long terminal repeat-associating protein 2 (HHLA2) is a recently discovered ligand of the B7 family, and was first described as a T cell co-inhibitory molecule (Cheng et al., 2017). It belongs to group III of the B7 family (Yan et al., 2019). The current study assesses *HHLA2* immunohistochemical expression lung Cancer. It differentiates between *HHLA2* expressions in different histopathological types. It also correlates the expression with other different clinicopathologic parameters. Thus, we can study *HHLA2 *impact on patient prognosis. It also could help clarify the value of *HHLA2* as potential target for immunotherapy in lung cancer. 

## Materials and Methods

The current study is retrospective study. It includes 62 biopsy specimens either bronchoscopic or CT-guided biopsies diagnosed as lung cancer in Oncology Center and the Clinical Oncology and Nuclear Medicine department, in collaboration with the Chest Medicine and Pathology departments, Mansoura University, Mansoura, Egypt during the period from 2016 to 2020. This work had the approval of institutional research board of Faculty of Medicine, Mansoura University (R.20.09.1026).

The cases were chosen randomly. Simple random sampling was performed using excel software to choose the final sample. Any chosen patient with absent paraffin block was replaced by another using the same method. The relevant clinicopathologic data was collected. Also, we followed the clinical outcome of the patients in the form of progression free survival (PFS) and overall survival (OS). Overall survival was calculated from diagnosis to death. PFS is the time from treatment initiation to 1st recurrence or metastasis or death. H&E slides of the pathologic specimens were reviewed to assess adequacy of tumor tissue for immunostaining.

Immunohistochemical Staining: Sections from formalin-fixed paraffin-embedded tissue blocks were deparaffinized and hydrated by standard procedures. Antibodies for HHLA2 (Rabbit polyclonal Ab, A13262, IgG, Abclonal, Inc) and EGFR (Rabbit polyclonal Ab, A11351 IgG, Abclonal, Inc) were utilized based on manufacturer instructions with appropriate positive control and negative control.

Immunohistochemical Assessment: slides were independently scored by 2 pathologists who had no knowledge about patients’ data. Positive *HHLA2 *expression was defined as >5% positive tumor cells (Yan et al., 2019). The intensity of HHLA2 staining was recorded as 0, 1, 2 and 3 for absent, mild, moderate and strong expression, respectively (Cheng et al., 2018). Positive EGFR expression was considered when staining > 1% tumor cells (Petersen et al., 2017). 

Statistical Analysis: Data was analyzed by SPSS software V.26. Categorical data was expressed as frequencies and percentages. Continuous data was shown as mean (SD) or Median (minimum-maximum) based on Shapiro-Wilk testing for the assumption of normal data distribution. Statistical significance testing of continuous data was done using Mann-Whitney U or Median Test. while, chi square test or Fisher’s Exact Test were used for categorical data, wherever appropriate. Spearman’s rho test was utilized to explore the correlation between *HHLA2* and *EGFR* markers. Kaplan-Meier test was utilized to assess OS and PFS of patients as regards tumor expression of *HHLA2*. Comparison was performed utilizing Log Rank (Mantel-Cox). Level of significance level was at 0.05. 

## Results

*Clinicopathological Characteristics of the Patients*

The study enrolled 62 lung carcinoma cases with median duration of follow up 7.6 months (min-max: 1-41 months). Their mean age was 60.3 years (SD: 14.4). About 84% of them were males, 8.1% were diabetic, 9.7% were hypertensive, and 6.9% of them had asthma. 50 cases (87.7%) had advanced tumor size (T3 or T4). At presentation, only 15 patients (27.2%) had no lymph node metastases. Also, 47 patients (83.9%) had metastases. For NSCLC, The most common pathological type was adenocarcinoma which was found in 26 cases (41.9%), followed by 15 casas squamous cell carcinoma (24.2%) and 14 cases SCLC (22.6). 38 cases (80.9%) were presented at stage IV. For SCLC, 9 patients (64.4%) were presented by extensive disease. 46 cases (74.2%) express *HHLA2* and about 21 positive cases (45.7%) showed mild expression of *HHLA2* compared to 20 cases (43.2%) with moderate expression and only 5 cases (8.1%) with high expression. EGFR was positive in 40 cases (64.5%) of including 33 cases (71.7%) of NSCLC compared to 5 cases (35.7 %) of SCLC, in addition to 2 cases of large cell neuroendocrine carcinoma (p=0.014).

*HHLA2 Expression in Relation to Other Clinicopathological Characteristics*

**Figure 1:**
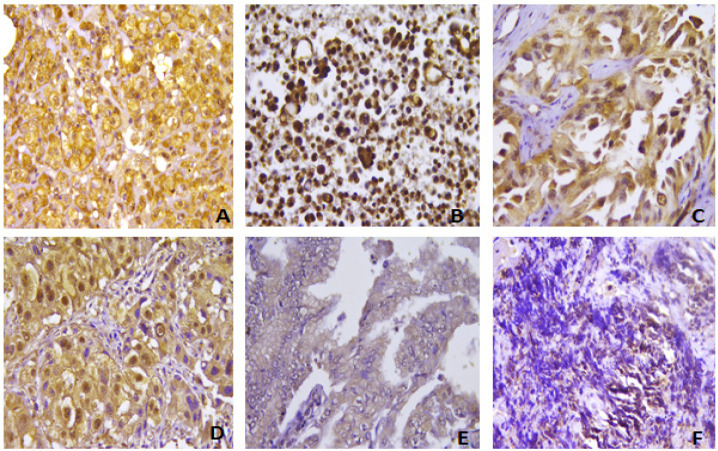
Immunohistochemical Staining of *HHLA2* in Different Cases of Lung Carcinoma: Strong expression in a case of squamous cell carcinoma (A) and in a case of papillary adenocarcinoma (B). Moderate *HHLA2* expression in a case of acinar adenocarcino

The association between *HHLA2* tumour expression and various parameters of the patients is presented in Table 1 and Figure 1. There was no statistically significant associations between age or sex and the *HHLA2* expression (p: 0.106, 0.709), respectively. Regarding the staging of the tumor, there was a significant association between* HHLA2* expression and metastasis. About 83% of patients presented with metastasis showed positive expression of *HHLA2* compared to 44.4% in patients with no metastasis (p=0.024). Among cases with SCLC, *HHLA2* expression was significantly higher in extensive stage (80%), compared to 0% of limited stage, (p=0.035). While, expression of *HHLA2* was higher in stage IV of NSCLC cases (86.5%), compared to stage II and III (44.4%) (p=0.006). The pathological type was not associated with *HHLA2* expression (p=0.488). Results show a significant mild positive correlation between expression of *HHLA2* and* EGFR* markers (r=0.256, p=0.045)

Table 2 shows the association between the intensity of positive *HHLA2* expression and the clinicopathologic characteristics of the patients. None of the Clinicopathologic parameters of the patients was associated with the intensity of the expression of *HHLA2*. There was a nonsignificant correlation between the intensity of expression of *HHLA2*, and *EGFR* (r= 0.307, p=0.087)

*Association between HHLA2 Expression and Survival*

A univariate analysis was done to assess the effect of *HHLA2* expression on survival. Kaplan-Meier survival curves were made, and then log-rank test was used.

**Figure 2.**
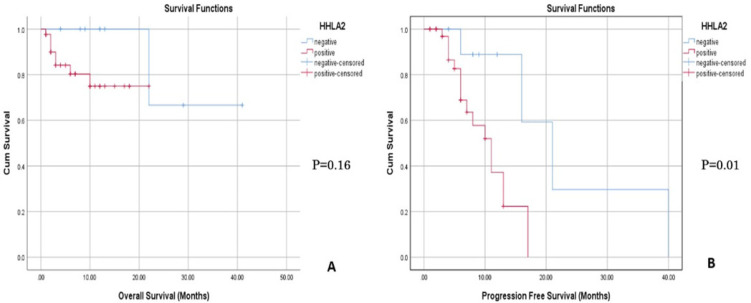
Analysis of Survival in Relation to Tumor Expression of HHLA2: (A) Overall survival (OS) and HHLA2: Positive expression was lower than patients with negative HHLA2 (p: 0.16) (B) Progression free survival (PFS) and HHLA2: PFS is lower among

Correlation of overall and progression-free survivals of cases and tumor expression of *HHLA2* marker are presented in Table 3, and Figure 2. The mean OS time in cases with positive *HHLA2* expression (17.6) was nearly half that of patients with negative expression of the markers (34.7). However, this difference was not statistically significant (p: 0.16). But, PFS of patients was significantly lower among the group with positive expression of *HHLA2* (10.3) compared to the group with negative expression of *HHLA2* (23.4) (p: 0.010). 

**Table 1:** The Association of Clinicopathologic Characteristics with the Expression of *HHLA2* (N=62).

Clinicopathologic Characteristics		Negative N (%) 16 (25.8)	Positive N (%) 46 (74.2)	Significance
Age (Ys)	Median (min-max)	67 (43-79)	59 (28-80)	Z=-1.6, p=0.106^a^
Gender	Male	13(25)	39(75)	P=0.709^b^
	Female	3(30)	7(70)	
Tumor (N=57)	T1/T2	0	7 (100)	P=0.186
	T3/T4	13 (26)	37 (74)	
Lymph nodes (N=55)	N0	3 (20)	12 (80)	P=1 ^b^
	N2/N3	10 (25)	30 (75)	
Metastasis (N=56)	M0	5 (55.6)	4 (44.4)	P= 0.024^b^
	M1	8 (17)	39 (83)	
Pathological types	SCLC	5 (35.7)	9 (64.3)	R
	NSCLC	10 (21.7)	36 (78.3)	P = 0.309 ^b^
	Large cell neuroendocrine carcinoma	1 (50)	1 (50)	P =1 ^b^
Stage SCLC	Limited	5 (100)	0	
	Extensive	1 (11.1)	8 (88.9)	P=0.035 ^b^
Stage NSCLC	II /III	5 (55.6)	4 (44.4)	P=0.006 ^b^
	IV	5 (13.5)	32 (86.5)	
EGFR expression	Negative	9 (40.9)	13 (59.1)	R=0.256*, p=0.045 ^b^
	Positive	7 (17.5)	33 (82.5)	

^a^, Mann-Whitney U; ^b^, Fisher's Exact Test; *, Spearman's rho

**Table 2:** The Association of Clinicopathologic Characteristics with the Intensity of Expression of *HHLA2* (N=46).

Clinicopathologic Characteristics		Mild N (%) 21 (45.7)	Moderate/ Strong N (%) 25 (54.3)	Significance
Age (Ys)	Median (min-max)	58 (31-72)	60.5(28-80)	Z= 1.1., p=0.267^a^
Gender	Male	20 (51.3)	19 (48.7)	P=0.106 ^b^
	Female	1 (14.3)	6 (85.7)	
Tumor (N=44)	T1/T2	2 (28.6)	5 (71.4)	P=0.428 ^b^
	T3/T4	18 (48.6)	19 (51.4)	
Lymph nodes (N=42)	N0	4 (33.3)	8 (66.7)	X^2^=0.96, p=0.327
	N2/N3	15 (50)	15 (50)	
Metastasis (N=43)	M0	1 (25)	3 (75)	P=0.610 ^b^
	M1	19 (48.7)	20 (51.3)	
Pathological types	SCLC	4 (44.4)	5 (55.6)	R
	NSCLC	16 (44.4)	20 (55.6)	p=1^ b^
	Large cell neuroendocrine carcinoma	1 (100)	0	P=1^ b^
Stage NSCLC	II/ III	1 (25)	3 (75)	P=0.613^ b^
	IV	15 (46.9)	17 (53.1)	
Positive EGFR	Mild	8 (61.5)	5 (38.5)	r= 0.307*, p=0.087
	Moderate/strong	13 (39.4)	20 (60.6)	

a, Mann-Whitney U; b, Fisher's Exact Test; *, Spearman's rho

**Table 3:** The Analysis of Survival in Relation to Tumor Expression of *HHLA* (Kaplan-Meier Test).

		Total N	N of events	Censored N (%)	Survival time Mean (95%CI)	p
Overall Survival	Negative	12	1	11 (91.7)	34.7 (24.5-44.8)	0.16
	Positive	43	8	35 (81.4)	17.6 (14.9-20.3)	
Progression Free Survival	Negative	4	4	8 (55.3)	23.4 (10.6-36.4)	0.01
	Positive	43	17	29 (58)	10.3 (9.5-18.1)	

*, Log Rank (Mantel-Cox)

## Discussion

Lung cancer remains the world’s major cause of mortality, and is one of the most frequently diagnosed cancers (Lin, 2019). Tumor immune evasion is one of the hallmarks of malignancy, and expression of the B7 family of ICPs Immune checkpoint molecules (*PD-L1, PD-L2, B7-H3, B7x* and *HHLA2*) is one mechanism of immune evasion by tumors. These molecules could suppress of T-cell function and proliferation and subsequently cytokine production (Flajnik et al., 2012; Zhao et al., 2013; Zhu et al., 2013; Janakiram et al., 2015b; Janakiram et al., 2017; Cheng et al., 2017). Given its emerging role of suppressing tumor immune responses with possible different immune evasion mechanisms from PD-1/PD-L1 pathways (Zang et al., 2010; Janakiram et al., 2012), there has been a great interest for exploration of *HHLA2* as a therapeutic immune target (Jeon et al., 2014; Janakiram et al., 2015b). Targeting these interactions through immunomodulation drugs has remarkable clinical success in several cancers (Inal et al., 2015).

In the current study, we examined immunohistochemical *HHLA2* expression in lung carcinoma specimens and its correlation to clinicopathological features. We studied *HHLA2* expression in NSCLC (adenocarcinoma, squamous cell carcinoma, and large cell undifferentiated carcinoma) and SCLC. *HHLA2* expression was found in 74% of the patients with tendency towards increased expression in *NSCLC* (78.3% of the positive cases). Similar to our results, Cheng et al., (2017) firstly reported high expression of *HHLA2* in lung cancer. It was positive in 71% of patients in a cohort study and also was expressed in two thirds (66%) of NSCLC cases (Cheng et al., 2017). In a more recent study by cheng et al, 2018, HHLA2 was positive in most of lung cancer specimens, including 61% of neoplasms in the discovery set and 64% in the validation cohort (Cheng et al., 2018).

In our study, there were no statistically significant associations between age or gender and the HHLA2 expression. This is consistent with what was previously reported in a study done by cheng et al., (2017) (Cheng et al., 2017). Also, we found no significant relationship between *HHLA2* expression and both tumor and lymph node stages. Similarly *HHLA2 *expression was not correlated to lymph node status in lung cancer (Cheng et al., 2017). But in triple-negative breast cancer (TNBC), overexpressed HHLA2 was linked to lymph node positivity (Janakiram et al., 2015a).

However, a statistically significant association was detected between* HHLA2* expression and both tumor staging and metastasis. Our results found that *HHLA2 *expression was higher in metastatic, stage IV disease than stage II and III disease. On the contrary, no association between *HHLA2* expression and various stages of lung cancer was revealed in the study by Cheng et al., (2017). But in TNBC, overexpressed *HHLA2* was linked to advanced stage of cancer at diagnosis, and also was linked to a high recurrence risk (Janakiram et al., 2015a). Also, *HHLA2* expression in osteosarcoma was reported to be associated with metastases (Koirala et al., 2016). 

In our study, *EGFR* was significantly expressed in NSCLC compared to SCLC (p=0.014). It is in agreement with previous studies which confirmed that EGFR mutations were one of the commonest oncogenic changes in NSCLC. Lung carcinoma cases with such mutations show distinct pathogenesis, clinical manifestations and disease course (Janakiram et al., 2016). 

As regards the Association of *HHLA2* and *EGFR*, results revealed that *EGFR* expression was significantly correlated with *HHLA2 *expression. Positive HHLA2 staining was found in 82.5% of cases with positive *EGFR* expression (P=0.045). This finding is in line with previous studies which reported that *EGFR* mutation was significantly correlated with overexpression of *HHLA2*. This indicates that *HHLA2* is a potentially novel target for lung cancer immunotherapy, especially in patients with *EGFR* expression. Also, this is particularly significant because *HHLA2* expression may assist identification of a group of patients who might do poorly in spite of tyrosine kinase inhibitors (TKIs) for *EGFR*-mutated tumours (Janakiram et al., 2016; Cheng et al., 2017; Cheng et al., 2018). 

Few studies were done on the prognostic value of *HHLA2* in lung cancer. In our study, *HHLA2 *overexpression with decreased survival was frequent. However this was not statistically significant with OS (p = 0.16). On the other side, a significant relation was detected with PFS (p = 0.01) , where patients with positive expression had shorter OS and PFS in comparison to patients with negative expression ( 17.6 vs 34.7 m, 10.3 vs 23.4 m) respectively. 

Our results are in line with a previous study which found that tumor *HHLA2* expression was significantly correlated with OS of NSCLC. In *EGFR* mutated tumors, there was a trend towards *HHLA2* overexpression with reduced OS (p = 0.19). However, this was not statistically significant (Cheng et al., 2017).

Many studies have reported that HHLA2 was a negative indicator in many malignancies as colon, lung and pancreatic malignancies (Cheng et al., 2017; Yan et al., 2019; Zhang et al., 2020; Zhu and Dong, 2018). The poor prognostic value of *HHLA2* was also reported in clear cell RCC, osteosarcoma and TNBC (Janakiram et al., 2015a; Koirala et al., 2016; Chen et al., 2018; Chen et al., 2019). However, high *HHLA2* expression was linked to better postsurgical prognosis in pancreatic and ampullary malignancies when utilizing a different anti-HHLA2 antibody clone for IHC (Boor et al., 2020). 

This indicates that the prognostic and clinical significance of *HHLA2* expression in different tumors might be complex and influenced by several factors (Chen et al., 2018). Novel immunotherapeutic approaches targeting *HHLA2* are under active development and have demonstrated hopeful results in many studies on other cancers. The wide expression of *HHLA2* in lung cancer implicates the therapeutic potential of targeting those immune markers also in lung cancer (Dangaj et al., 2013; Janakiram et al., 2017). 

Our study had some limitations since it is a retrospective one and other etiological factors including smoking and therapeutic factors following relapse could affect OS. Other factors influencing *HHLA2* expression such as T- cell subsets, expression of other co-inhibitory molecules as *PD-L1, B7-H3* and *B7x* were not assessed. Further research is required to determine the biological significance and detect mechanisms of high *HHLA2* expression in lung cancer and clarify its contributions in tumor immune escape.

In conclusions, This study suggests that recently discovered, *HHLA2* is over expressed in lung cancer associating with higher stage. It is also correlated with *EGFR* overexpression. *HHLA2* could serve as a predictor of progression and distant metastasis. Also, it has potential to be effective immune target in lung cancer immunotherapy such as checkpoint blockade and antibody-drug conjugate treatment.

## Author Contribution Statement

Author 1: Concepts, Design, Literature search, Data analysis, Manuscript editing& review Authors 2, 3, 6: Clinical Studies and Data acquisition. Author 4: Data & Statistical analysis and manuscript review.

MF put the research design, did the literature search, performed the histological examination, analyzed data, wrote & reviewed the manuscript. EI performed the histological examination. TE, MA, AE & HA acquired the clinical data and did the clinical studies. HA wrote and reviewed the manuscript. All authors read and approved the final manuscript.
